# Bisphenol A Impairs Hepatic Glucose Sensing in C57BL/6 Male Mice

**DOI:** 10.1371/journal.pone.0069991

**Published:** 2013-07-29

**Authors:** Leigh Perreault, Carrie McCurdy, Anna A. Kerege, Julie Houck, Kristine Færch, Bryan C. Bergman

**Affiliations:** 1 Division of Endocrinology, Metabolism and Diabetes, University of Colorado Anschutz Medical Campus, Aurora, Colorado, United States of America; 2 Department of Pediatrics, University of Colorado Anschutz Medical Campus, Aurora, Colorado, United States of America; 3 Steno Diabetes Center, Gentofte, Denmark; Universidad Miguel Hernández de Elche, Spain

## Abstract

**Aims/Hypothesis:**

Glucose sensing (eg. glucokinase activity) becomes impaired in the development of type 2 diabetes, the etiology of which is unclear. Estrogen can stimulate glucokinase activity, whereas the pervasive environmental pollutant bisphenol A (BPA) can inhibit estrogen action, hence we aimed to determine the effect of BPA on glucokinase activity directly.

**Methods:**

To evaluate a potential acute effect on hepatic glucokinase activity, BPA in water (n = 5) vs. water alone (n = 5) was administered at the EPA’s purported “safe dose” (50 µg/kg) by gavage to lean 6-month old male C57BL/6 mice. Two hours later, animals were euthanized and hepatic glucokinase activity measured over glucose levels from 1–20 mmol/l in liver homogenate. To determine the effect of chronic BPA exposure on hepatic glucokinase activity, lean 6-month old male C57BL/6 mice were provided with water (n = 15) or water with 1.75 mM BPA (∼50 µg/kg/day; n = 14) for 2 weeks. Following the 2-week exposure, animals were euthanized and glucokinase activity measured as above.

**Results:**

Hepatic glucokinase activity was signficantly suppressed after 2 hours in animals given an oral BPA bolus compared to those who received only water (p = 0.002–0.029 at glucose 5–20 mmol/l; overall treatment effect p<0.001). Exposure to BPA over 2 weeks also suppressed hepatic glucokinase activity in exposed vs. unexposed mice (overall treatment effect, p = 0.003). In both experiments, the Hill coefficient was higher and Vmax lower in mice treated with BPA.

**Conclusions/Interpretation:**

Both acute and chronic exposure to BPA significantly impair hepatic glucokinase activity and function. These findings identify a potential mechanism for how BPA may increase risk for diabetes.

## Introduction

Weight loss and exercise remain the cornerstones of diabetes prevention. Nevertheless, the paradigm that dietary over-consumption and physical inactivity are the single determinants of weight gain and diabetes risk has recently been challenged as overly simplistic. In 2002, Baillie-Hamilton postulated a role for chemical toxins in the etiology of obesity by showing that the obesity epidemic coincided with the marked increase of industrial chemicals in the environment over the past 40 years [1]. The global hypothesis put forth by Baillie-Hamilton was that environmental toxins contribute to obesity through dysregulated feeding behavior. To date, this hypothesis is unproven, but has sparked considerable interest in the role of pesticides, organic pollutants and environmental toxins in human health and disease. Indeed, a recently published post hoc analysis from the 2003–2004 U.S. National Health and Nutrition Examination Survey (NHANES) showed a 39% increase in incident type 2 diabetes for every 1 standard deviation increase in urinary concentration of the environmental toxin, bisphenol A (BPA) [2]. Importantly, the 4 other phenols measured did not correlate with diabetes incidence or any other disease. The authors concluded that BPA conferred particular risk for diabetes which is especially concerning given the widespread human exposure.

Proposed mechanisms linking BPA to the development of diabetes are numerous and include fetal programming [3], sex steroid imbalance [4], altered adipocyte biology [5], disrupted beta cell function [6] and oxidative stress [7]. Nevertheless, because BPA is hepatically metabolized, its toxicity in the liver appears particularly prominent [7]. Further, there is reason to speculate that BPA may be an environmental inhibitor of hepatic glucokinase, specifically. Glucokinase has been definitively shown as the body’s glucose sensor in beta cells, enteroendocrine cells of the gut, hypothalamus and liver. Retinoic acid [8], [9], biotin [10], [11], and sorbitol [12] have all been shown to alter the transcription, translation, or activity of glucokinase, but none of these factors have changed much in the American diet over the past 30 years, making them unlikely candidates as dietary regulators of diabetes risk. In contrast, BPA – new to the American diet over the past 40 or so years – is also known as an “endocrine disruptor” due its estrogenic effects [6], [13]–[15]. The latter is of particular interest as estrogen action may be antagonized, not synergized, by BPA specifically in the liver [16]–[18] where glucokinase has a key glucoregulatory role. Despite the known actions of estrogen on glucokinase, and of BPA on estrogen action, no study to date had examined BPA’s effect on glucokinase, hence was the aim of the current investigation.

## Methods

### Experimental Design

All animal studies were approved by the Institutional Animal Care and Use Committee at the University of Colorado School of Medicine. All studies were conducted in lean male C57BL/6 mice 6 months of age.

#### Acute BPA exposure

To determine the acute effect on hepatic glucokinase activity, BPA in water (n = 5) vs. water alone (n = 5) was administered at the Environmental Protection Agency’s (EPA) purported “safe dose” (50 µg/kg) by gavage. Two hours later, animals were anesthetized with isoflurane and euthanized by cervical dislocation. Livers were rapidly dissected, weighed and immediately homogenized for the measurement of hepatic glucokinase activity.

#### Chronic BPA exposure

To determine the chronic effect of BPA on hepatic glucokinase activity, mice were provided ad libitum access to water without (n = 15) or water with 1.75 mM BPA in glass bottles which resulted in an estimated dose of ∼50 µg/kg/day for 2 weeks (n = 14). BPA concentration was measured in the urine of the animals at day 14 to confirm exposure. Animals were euthanized at day 14, livers collected and glucokinase activity measured, as described below.

### Analytical Procedures

#### Hepatic glucokinase activity

Hepatic glucokinase (hexokinase IV) activity was determined in whole liver homogenate using a colorimetric assay (absorbance at 340 nm for 20 minutes; BioTek Synergy H1 hybrid reader, using Gen5 v2.00 software). Livers were weighed and homogenized for 1 minute using a Kinble Contes glass homogenizer in a buffer containing 100 mM Tris HCl, then adjusted to a final 10X dilution. Ten µl of the homogenate was used for the assay. In a separate plate, pre-mixed cocktail solutions containing different concentrations of glucose were aliquotted, then transferred to the wells containing liver homogenate. This was done over glucose concentrations 0–20 mmol/l. Glucose 6-phosphate formed by glucokinase was measured by the formation of NADPH in the presence of G6PDH, as previously described [19].

#### Urine samples

Urinary BPA concentration was measured using a super-sensitive ELISA (Biosense Laboratories; Bergen, Norway).

### Calculations

The average mouse weight was 29.2 g and the average water intake was 8 ml/day. For the 2-week exposure protocol, BPA was dissolved in water to 1.75 mM resulting in an average daily exposure of 50 µg/kg/day. One unit of glucokinase activity is defined as the amount of the enzyme required to produce 1 µmol of G-6-P in 1 minute. Reported data are for specific activity of the enzyme (activity/protein concentration).

### Statistical Analysis

Data are presented as mean ± SEM. Glucokinase activity over a range of glucose concentrations were examined for best fit prior to analysis (GraphPad Prism 5; La Jolla, CA). An overall treatment effect in BPA-exposed vs. non-exposed mice was estimated by repeated measures (two-way ANOVA). Differences in glucokinase activity between groups for a given glucose concentration were compared using two-sample Student’s t-tests. Negative values for glucokinase activity were considered zero. An alpha level of 0.05 was used throughout the study.

## Results

### Acute BPA Exposure

**Figure 1:**
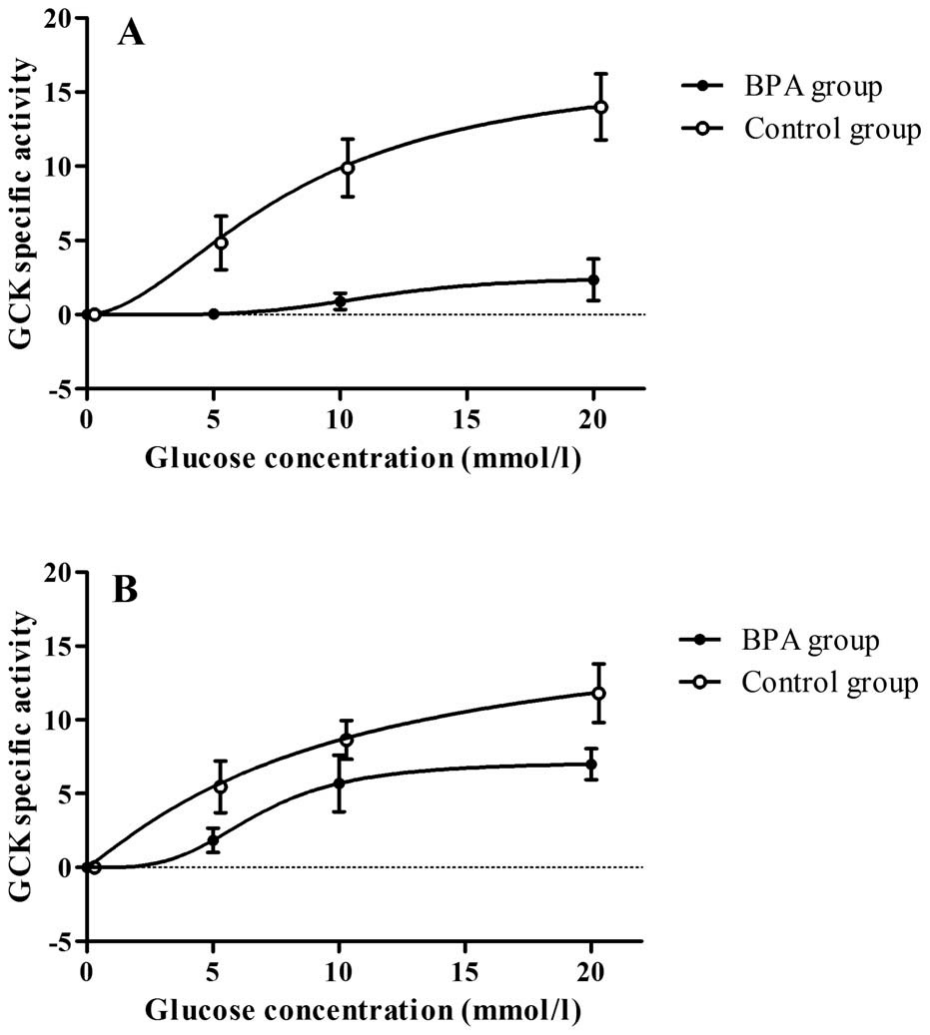
Glucokinase specific activity at different glucose concentrations (range 0–20 mmol/l) in mice 2 hours after acute BPA exposure (A; overall treatment effect p<0.001) or after 2 weeks chronic BPA exposure (B; overall treatment effect p = 0.003).

Hepatic glucokinase kinetics for the BPA-treated and control mice are described in Table 1. Glucokinase activity was significantly lower at 5 mmol glucose for the BPA exposed animals (p = 0.029), as well as at 10 mmol/l (p = 0.002) and 20 mmol/l (p = 0.002) glucose. As depicted in Figure 1A, 2-hour BPA exposure largely abolished glucokinase activity over the range of physiologic glucose concentrations tested (overall treatment effect p<0.001) through a decline in the Vmax and an increase in the enzyme’s Hill coefficient.

**Table 1:** Glucokinase (GCK) kinetics in the control and BPA groups after acute and chronic BPA exposure.

ACUTE EFFECT OF BPA			
	Control group	BPA group	P value
N	5	5	
Mouse weight (g)			
GCK activity (µM/min(•10^3^) at glucose concentrations:			
5 mmol/L	4.84 (1.80)	0.00 (0.00)	0.029
10 mmol/L	9.88 (1.93)	0.89 (0.55)	0.002
20 mmol/L	14.00 (2.22)	2.35 (1.40)	0.002
Overall treatment effect			<0.001
Allosteric model fit	Perfect fit	Perfect fit	
Vmax	16.72	2.55	
Hill coefficient	1.83	4.46	
**CHRONIC EFFECT OF BPA**			
	**Control group**	**BPA group**	**P value**
**N**	**15**	**14**	
Mouse weight (g)			
Urinary BPA (µg/L)	1.0 (1.0)	22.0 (6.0)	0.02
GCK activity (•10^3^) at glucose concentrations:			
5 mmol/L	5.47 (1.74)	1.85 (0.81)	0.080
10 mmol/L	8.63 (1.31)	5.70 (1.91)	0.232
20 mmol/L	11.80 (1.97)	6.99 (1.05)	0.047
Overall treatment effect			0.003
Allosteric model fit	Perfect fit	Perfect fit	
Vmax	17.35	7.15	
Hill coefficient	1.10	3.49	

Data are means (SEM).

### Chronic BPA Exposure

Mice treated for 2 weeks with BPA had higher concentration of BPA in their urine compared to control animals (p = 0.02; Table 1), confirming their exposure. After 2 weeks of dietary BPA intake, hepatic glucokinase activity was significantly reduced over the range of glucose concentrations tested (overall treatment effect p = 0.003; Figure 1B) with particular divergence at 20 mmol/l (p = 0.047) and altered enzyme kinetics.

## Discussion

The rise in human exposure to bisphenol A [2,2-bis(p-hydroxylphenyl)propane] (BPA) has largely mirrored the rise in incident type 2 diabetes. BPA is a chemical used widely in the production of epoxy resins and polycarbonate plastics. Because of its pervasive use in the lining of food and beverage containers, medical supplies and dental sealants, human exposure has been deemed virtually ubiquitous. Population biomonitoring studies estimate the current adult exposure to BPA ≤1 µg/kg/day [20], well below the purported “safe dose” of 50 µg/kg/day set by the Environmental Protection Agency in the 1980’s. Yet few assertions in medicine today are as controversial. A recent review of the >80 biomonitoring studies cast serious doubt on whether past estimates are accurate or whether the acceptable level of exposure ensures any degree of safety [21]. Because of BPA’s known anti-estrogenic effects [7] and the known effect of estrogen to stimulate glucokinase [22] – the body’s glucose sensor – we examined the *direct* effect of BPA on hepatic glucokinase activity. Major findings from this study demonstrate that both acute and chronic BPA exposure – at a dose deemed safe in humans –impair hepatic glucokinase activity and function. These results provide a potential link as to how BPA exposure may increase risk for diabetes and have alarming implications for public health.

To explore the association between BPA exposure and increased risk for diabetes, we examined the effect of acute ingestion of BPA on glucose sensing. Orally administered BPA is metabolized by glucuronidation during first pass metabolism in the liver, with its peak seen at 2 hours, a biological half-life of approximately 6 hours and nearly complete elimination in 24 hours [23]. Based on the above BPA absorption parameters established in humans, we administered the maximum “safe dose” (50 µg/kg) dissolved in water to mice expecting maximum biologic effects 1–3 hours post-ingestion. Results from the current study demonstrate nearly complete suppression in overall hepatic glucokinase activity and significant alteration in intrinsic enzyme kinetics in mice 2 hours after ingestion of BPA. Although human exposure is unlikely to occur at this level in a single dose, these data are the first to contend that this dose is not safe and has physiological ramifications relevant to the development of diabetes, at least in mice.

Due to possible exposure through dermal absorption, inhalation and ingestion, the precise pharmacokinetics of chronic BPA exposure are less clear [24]. What is clear is that BPA exposure is virtually ubiquitous and constant. A recent survey identified BPA in 63 of 105 food samples, including fresh turkey, canned green beans, and infant formula [25], suggesting that human exposure is frequent, but also at low levels, rather than in large boluses. Ad libitum water (spiked with 1.75 mmol BPA) intake by our mice over 14 days also demonstrated a significant negative effect on overall hepatic glucokinase activity and function, albeit less pronounced than the effect of the acute BPA exposure. Importantly, however, these experiments capture the influence of low and intermittent exposure of BPA on hepatic glucose sensing, likely more representative of its true effects in the human population. Furthermore, mathematical models [26] would contend that impaired glucose sensing to the degree observed in the 2-hour and 2-week experiments could lead to clinical diabetes [27].

Several limitations of the current study are worth noting in its interpretation. First, experiments were conducted exclusively in mice, hence results may not be reproducible in humans. Second, all mice were male, hence we cannot comment on the impact of BPA exposure in female mice. Lastly, the effect of BPA exposure on glucokinase activity was presumed through the action of BPA on the estrogen receptor, however, this was not specifically investigated in this brief report.

In conclusion, major findings from this study demonstrate that both acute and chronic BPA exposure – at a dose deemed safe in humans –impair hepatic glucokinase activity and function in mice. These results provide a plausible link between BPA exposure and risk for diabetes. Future research is needed to determine whether BPA impairs glucose sensing in humans and if it can be restored through BPA avoidance. Such research could have a significant impact on food packaging, dietary selections and legislation relevant to public health.
